# Serum metabolite profiles as potential biochemical markers in young adults with community-acquired pneumonia cured by moxifloxacin therapy

**DOI:** 10.1038/s41598-020-61290-x

**Published:** 2020-03-10

**Authors:** Bo Zhou, Bowen Lou, Junhui Liu, Jianqing She

**Affiliations:** 1 Respiratory and Critical Care Medicine, The First Affiliated Hospital of Xi’an Jiaotong University, Xi’an, 710048 China; 2 Cardiology Department, The First Affiliated Hospital of Xi’an Jiaotong University, Xi’an, 710048 China; 3 Diagnostic Department, The First Affiliated Hospital of Xi’an Jiaotong University, Xi’an, 710048 China; 4 Key Laboratory of Environment and Genes Related to Diseases, Ministry of Education, Xi’an, 710048 China

**Keywords:** Biomarkers, Respiratory tract diseases

## Abstract

Despite the utilization of various biochemical markers and probability calculation algorithms based on clinical studies of community-acquired pneumonia (CAP), more specific and practical biochemical markers remain to be found for improved diagnosis and prognosis. In this study, we aimed to detect the alteration of metabolite profiles, explore the correlation between serum metabolites and inflammatory markers, and seek potential biomarkers for young adults with CAP. 13 Eligible young mild CAP patients between the ages of 18 and 30 years old with CURB65 = 0 admitted to the respiratory medical department were enrolled, along with 36 healthy participants as control. Untargeted metabolomics profiling was performed and metabolites including alcohols, amino acids, carbohydrates, fatty acids, etc. were detected. A total of 227 serum metabolites were detected. L-Alanine, 2-Hydroxybutyric acid, Methylcysteine, L-Phenylalanine, Aminoadipic acid, L-Tryptophan, Rhamnose, Palmitoleic acid, Decanoylcarnitine, 2-Hydroxy-3-methylbutyric acid and Oxoglutaric acid were found to be significantly altered, which were enriched mainly in propanoate and tryptophan metabolism, as well as antibiotic-associated pathways. Aminoadipic acid was found to be significantly correlated with CRP levels and 2-Hydroxy-3-methylbutyric acid and Palmitoleic acid with PCT levels. The top 3 metabolites of diagnostic values are 2-Hydroxybutyric acid(AUC = 0.90), Methylcysteine(AUC = 0.85), and L-Alanine(AUC = 0.84). The AUC for CRP and PCT are 0.93 and 0.91 respectively. Altered metabolites were correlated with inflammation severity and were of great diagnostic value for CAP.

## Introduction

Community-acquired pneumonia (CAP), the most common type of pneumonia, is one of the leading causes of mortality and morbidity worldwide^1^. Typical bacterial pathogens that cause CAP include *Streptococcus pneumoniae, Haemophilus influenzae*, and *Moraxella catarrhalis*^2,3^. The most common type of bacteria is *Streptococcus pneumoniae*, especially in young patients with CAP who could be cured by moxifloxacin^2,3^. The clinical presentation of CAP varies from mild pneumonia characterized by fever and productive cough to severe pneumonia characterized by respiratory distress and sepsis^4^. Although considerable progress has been made in understanding the molecular mechanisms underlying pulmonary infection, satisfactory diagnosis and treatment modalities remain limited^3^.

To date, a large number of biochemical markers have been explored as potential diagnostic variables for CAP, such as C-reactive protein (CRP) level, neutrophil percentage, and white blood cell count (WBC)^5^. Despite the utilization of various biochemical markers and probability calculation algorithms based on clinical studies of CAP, more specific and practical biochemical markers remain to be found for improved CAP diagnosis and prognosis prediction.

It has been proven that metabolic dysregulation could be detected in the peripheral blood of patients with CAP^6–9^. Studies about serum metabolism dysregulation have also provided evidence for a better understanding of the pathophysiological mechanism of pulmonary infectious disease^6,7^. However, most of these studies focused on alteration of metabolic profiles in severe pneumonia with respiratory distress or sepsis^7,10^, while few studies have investigated metabolic dysregulation in young patients with CAP, which leads to improved clinical outcomes but also requires early accurate diagnosis and timely treatment to prevent devastating complications. As a result, elucidating metabolic profiles and identifying potential novel circulatory markers based on a global metabolomics perspective might provide new evidence for improved diagnosis and treatment of young CAP patients.

In this study, by investigating the metabolite profiles in the serum of young patients with CAP compared to those in the serum of the respective controls, we aimed to detect the alteration of metabolite profiles, explore the correlation between serum metabolites and inflammatory markers, and seek potential biomarkers for young CAP patients. The primary aim of this study was to define altered metabolic profiles that clearly differentiated CAP patients and controls, with a secondary endpoint of identifying metabolic markers that help to diagnose CAP.

## Materials and Methods

### Subjects

All subjects were enrolled from the respiratory and critical care department of the First Affiliated Hospital of Xi’an Jiaotong University, China, from September 2017 to September 2018. Written informed consent was obtained from all subjects according to the Declaration of Helsinki, and the study was approved by the ethics committee of Xi’an Jiaotong University^11–13^. We included a cohort of 13 young adults and 36 healthy controls between the ages of 18 and 30 years old. The inclusion criteria were as follows. (1) Eligible young adults with CAP admitted to the respiratory medical department who met the diagnostic criteria for CAP(a constellation of suggestive clinical features, a demonstrable infiltrate by chest radiograph or other imaging technique, with or without supporting microbiological data). According to the CURB-65 criteria, the scores of the patients with CAP were all 0. (2) Potential subjects were between the ages of 18 and 30 years old. (3) Patients were cured and discharged from the hospital after moxifloxacin therapy for 1 week. The subjects were excluded if they (1) were taking or had previously taken any drugs known to influence lipid metabolism or the endocrine system; (2) had chronic respiratory diseases, cardiac disease or endocrine diseases; (3) were undergoing haemodialysis for renal failure; (4) had acute or chronic hepatitis with increased transaminase activities; (5) had a malignant tumour; or (6) were positive for IgM for influenza virus, parainfluenza virus, respiratory syncytial virus, adenovirus, *Legionella pneumophila*, mycoplasma or chlamydia. The patients were treated on an inpatient basis. Demographic and biochemical information was obtained as previously described^12–14^.

### Serum sample preparation and biochemical measurements

Serum samples were collected from young adults with CAP and control participants upon initial diagnosis or enrolment before moxifloxacin therapy. Venous blood was withdrawn after an overnight fast. Biochemical measurements, including complete blood count, CRP, procalcitonin (PCT) and liver and kidney function, were measured afterwards for participant inclusion and exclusion. The withdrawn blood samples were centrifuged at 3000 rpm for 10 min at 4 °C. Serum was separated and stored at −80 °C, and aliquots were thawed for further processing as previously described^12,13^.

### Pathogen detection

23.08%(3/13) young CAP enrolled in the present study display gram-positive cocci infection for sputum smear; the rest negative for the sputum culture and blood culture. Moreover, the subjects were excluded if they display positive IgM result for influenza virus, parainfluenza virus, respiratory syncytial virus, adenovirus, *Legionella pneumophila*, mycoplasma or chlamydia.

### Serum metabolism profile determination

Samples were thawed in an ice bath to diminish sample degradation. Each 100-µL aliquot of serum was spiked with two internal standards and mixed with 300 µL of an organic mixture for protein precipitation. All the standards were accurately weighed and prepared in water, methanol, sodium hydroxide solution, or hydrochloric acid solution to obtain individual stock solutions at a concentration of 5.0 mg/mL. The mixture was then vortexed for 30 s, stored at −20 °C for 10 min, and centrifuged for 10 min. The supernatant was transferred and dried in a 500-µL glass tube under vacuum conditions. After dissolving the dried materials, the derivative was injected into a mass spectrometer. Afterwards, quality control samples, which were prepared by mixing equal amounts of serum samples from all enrolled subjects, were used to control intergroup variability. For the detection of metabolites, we utilized a gas chromatography time-of flight mass spectrometry (GC-TOF/MS) system (Pegasus HT, Leco Corp., St. Joseph, MO, USA) with an Agilent 7890B gas chromatograph and a Gerstel multipurpose MPS2 sampler with dual heads (Gerstel, Muehlheim, Germany). Metabolites including alcohols, aldehydes, alkylamines, amino acids, carbohydrates, fatty acids, hormones, indoles, lipids, nucleotides, organic acids, phenols and vitamins were detected^15–17^. Samples were randomized prior to the XploreMET platform to decrease experimental bias and drift. Untargeted metabolomics profiling was performed on the XploreMET platform (Metabo-profileTM, Shanghai, China)^12,13^. The ratios between correlated metabolites with biological significance were also calculated to explore potential dysregulated enzymes and metabolic pathways in young adults with CAP.

### Statistical analysis

Data were normalized using MetaboAnalyst before analyses as previously described^18–27^ (Supplementary Fig. S1). Partial least squares discriminant analysis (PLS-DA) models were employed to reduce a large number of correlated metabolites to a smaller number of uncorrelated factors, and variable importance in projection (VIP) measurements of dysregulated metabolites were calculated to explain the importance of differentially abundant metabolites. Enrichment analysis was performed based on pathway-associated and metabolite sets^28,29^. The correlation and regression analysis of metabolites and clinical data was performed using Pearson’s correlation and simple linear regression (SPSS 20.0). Receiver operating characteristics (ROCs) were used, and areas under the ROC curve (AUC) were calculated to explore the discriminative capability of different metabolites or ratios to identify young adults with CAP. Categorical variables are presented as percentages, and continuous variables are presented as the mean ± SE. FDR-adjusted p-values <0.05 were considered significant.

### Ethics approval and consent to participate

Written informed consent was obtained from all study participants, with ethnic committee approval at the First Affiliated Hospital of Xi’an Jiaotong University.

## Results

### Clinical characteristics of the subjects enrolled

Previous studies have investigated the metabolic profile in patients with severe pulmonary infection and sepsis, but few studies have explored metabolic changes in young adults with mild pneumonia^7,10^. To address this question, after an initial screen from more than 2000 inpatients and outpatients diagnosed with CAP, we enrolled 13 young CAP patients who met the inclusion and exclusion criteria. Thirty-six healthy participants were enrolled as controls. Baseline factors were collected and are listed in Table 1. The mean age was 23.23 ± 2.20 years in the young CAP patient group and 25.03 ± 2.98 years in the control group. No significant differences were observed in sex and age, while the two groups exhibited different WBC counts, neutrophil percentages (%), and CRP and PCT levels, which are important markers for diagnosis and evaluation of the severity of infection (Table 1).

**Table 1:** Clinical characteristics of the patients with CAP and normal control.

Characteristics	Infection	Control	P
Age(years)	28.62 ± 8.17	31.14 ± 10.11	ns
Female	0.31%	0.50%	
WBC(*10^9/L)	12.14 ± 5.06	6.20 ± 1.78	<0.01
NEUT%(%)	85.82 ± 9.49	59.47 ± 9.64	<0.01
CRP(mg/L)	130.13 ± 91.66	2.02 ± 2.63	<0.01
PCT(ug/L)	5.30 ± 9.75	0.15 ± 0.31	<0.01

### The serum metabolic profile displayed differential metabolic features in young CAP patients compared to those in normal controls

**Figure 1:**
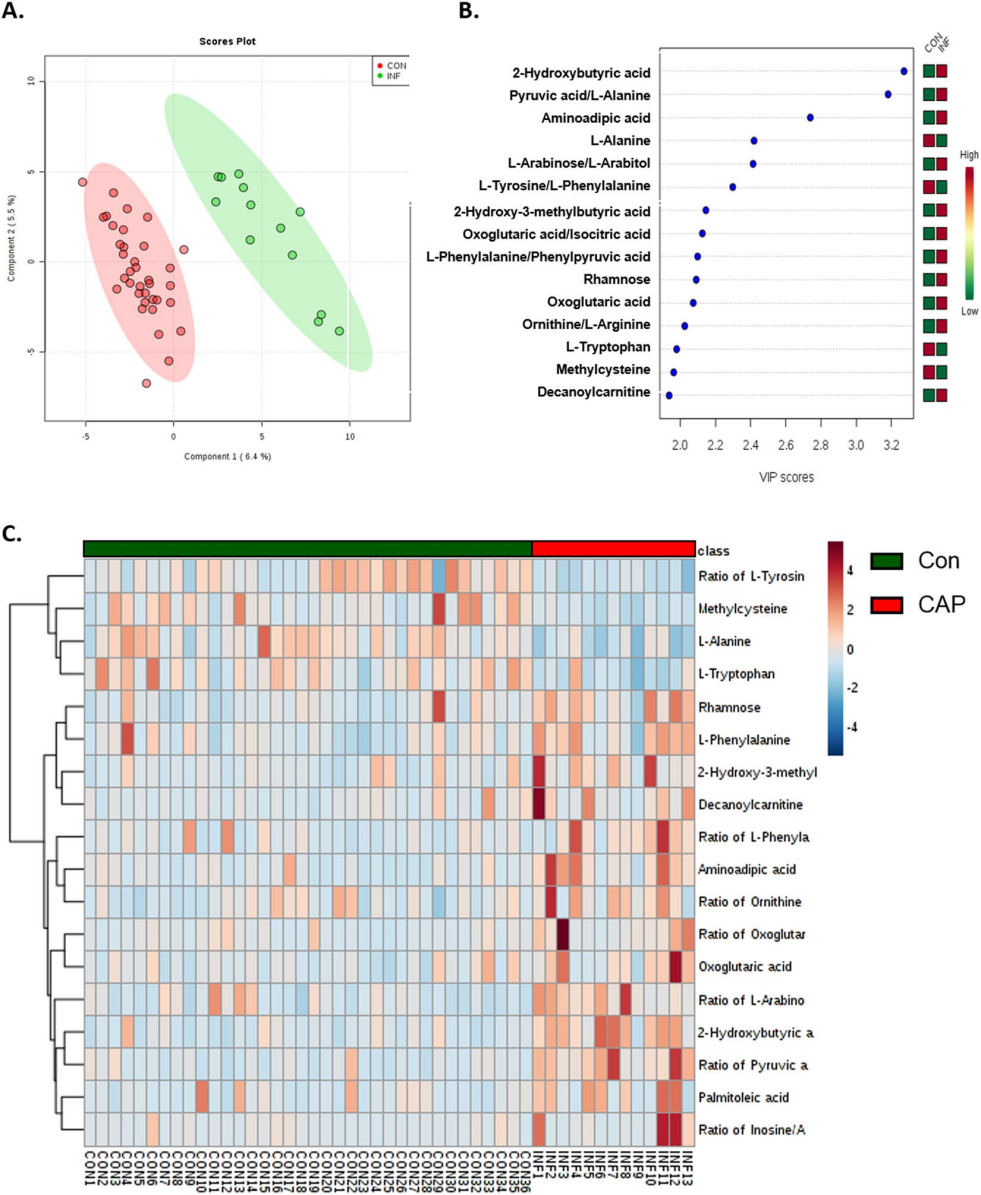
Serum metabolic profile displayed differential metabolic features in young adults with CAP as compared to normal control. (**A**) 2D projection plots of metabolites from PLS-DA analysis, indicating clear separation between the young CAP and control. (**B**) Variable importance in projection (VIP) of metabolites and their ratios of biological significance. (**C**) Heatmap of the significantly altered metabolites and their ratios of biological significance in the young CAP and control.

To further investigate the global metabolic profile alteration in young CAP patients compared to that in the controls, we first utilized PLS-DA models to reduce a large number of correlated metabolites to a smaller number of uncorrelated factors. There was a cleared separation of young CAP patients and the respective controls, as shown by PLS-DA in Fig. 1A. The VIP measurements of PLS-DA were calculated and displayed 2-Hydroxybutyric acid, Aminoadipic acid, L-Alanine, 2-Hydroxy-3-methylbutyric acid, Rhamnose, Oxoglutaric acid, L-Tryptophan, Methylcysteine, Decanoylcarnitine as important dysregulated metabolites, and Pyruvic acid/L-Alanine, L-Arabinose/L-Arabitol, L-Tyrosine/L-Phenylalanine, Oxoglutaric acid/Isocitric acid, L-Phenylalanine/Phenylpyruvic acid, Ornithine/L-Arginine as primary altered ratios (Fig. 1B).

**Figure 2:**
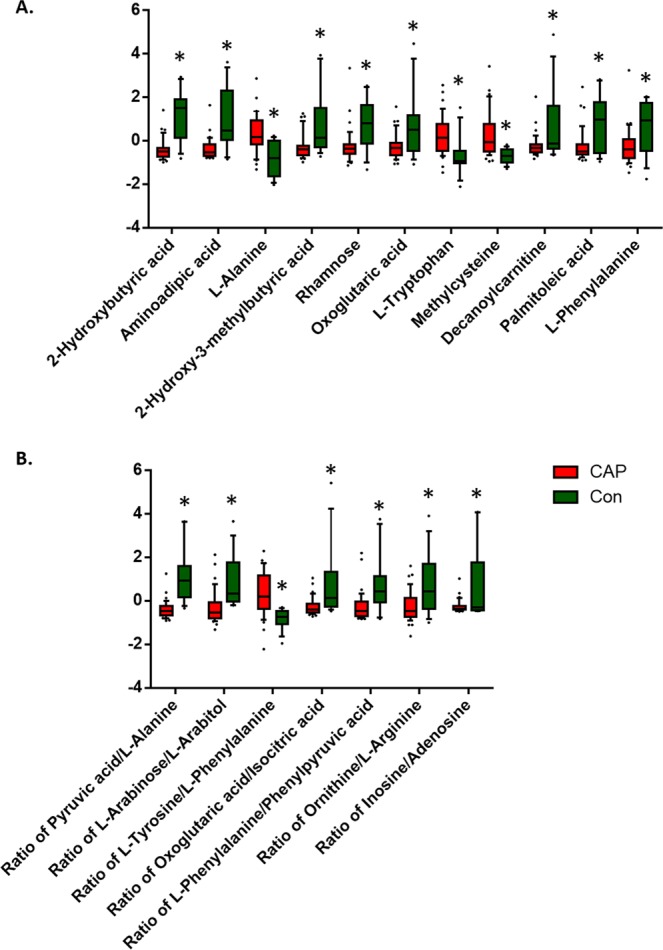
Metabolite levels were dysregulated in young adults with CAP as compared to control. (**A**) Relative levels of significantly altered metabolites in the young CAP and control. (**B**) Relative levels of significantly altered ratios of biological significance in the young CAP and control. Data were analyzed using Student t-test. Mean ± s.e.m. *p < 0.05.

A total of 227 serum metabolites, including alcohols, amino acids, carbohydrates and fatty acids, were detected (Supplementary Fig. 2A). We first analysed the overall metabolite class values between young CAP patients and the controls, which yielded no statistical significance (Supplementary Fig. 2B). We compared metabolite levels between young CAP patients and controls using Student’s t-test. An overview of significantly altered metabolites and ratios with biological significance is shown in Fig. 1C. Among them, L-Alanine, 2-Hydroxybutyric acid, Methylcysteine, L-Phenylalanine, Aminoadipic acid, L-Tryptophan belonging to amino acids, Rhamnose to carbohydrates, Palmitoleic acid to fatty acids, Decanoylcarnitine to lipids, and 2-Hydroxy-3-methylbutyric acid and Oxoglutaric acid to organic acids were found to be significantly altered, which correlates to the VIP measurements in PLS-DA (Fig. 2A, Table 2). Of note, metabolite ratios, including Pyruvic acid/L-Alanine, L-Arabinose/L-Arabitol, Tyrosine/L-Phenylalanine, Oxoglutaric acid/Isocitric acid, L-Phenylalanine/Phenylpyruvic acid, Ornithine/L-Arginine, Inosine/Adenosine ratios, were also significantly altered, indicating underlying biological pathway changes (Fig. 2B, Table 2).

**Table 2:** List of the differential metabolites and ratio.

	p.value	FDR
**Differential metabolites**		
2-Hydroxybutyric acid	1.41E-08	3.23E-06
Aminoadipic acid	7.60E-06	0.00057997
L-Alanine	0.0001173	0.0056116
2-Hydroxy-3-methylbutyric acid	0.0007981	0.02582
Rhamnose	0.0011275	0.02582
Oxoglutaric acid	0.0012499	0.02602
L-Tryptophan	0.0021843	0.038478
Methylcysteine	0.0024031	0.039308
Decanoylcarnitine	0.002775	0.042365
Palmitoleic acid	0.0032038	0.045854
L-Phenylalanine	0.0038732	0.049276
**Differential metabolites ratio**		
Ratio of Pyruvic acid/L-Alanine	5.03E-08	5.75E-06
Ratio of L-Arabinose/L-Arabitol	0.00012252	0.0056116
Ratio of L-Tyrosine/L-Phenylalanine	0.0002864	0.010931
Ratio of Oxoglutaric acid/Isocitric acid	0.00090572	0.02582
Ratio of L-Phenylalanine/Phenylpyruvic acid	0.0010714	0.02582
Ratio of Ornithine/L-Arginine	0.0016683	0.031836
Ratio of Inosine/Adenosine	0.0035215	0.047437

### Enrichment analysis showed significantly altered metabolic pathways in young CAP patients

**Figure 3:**
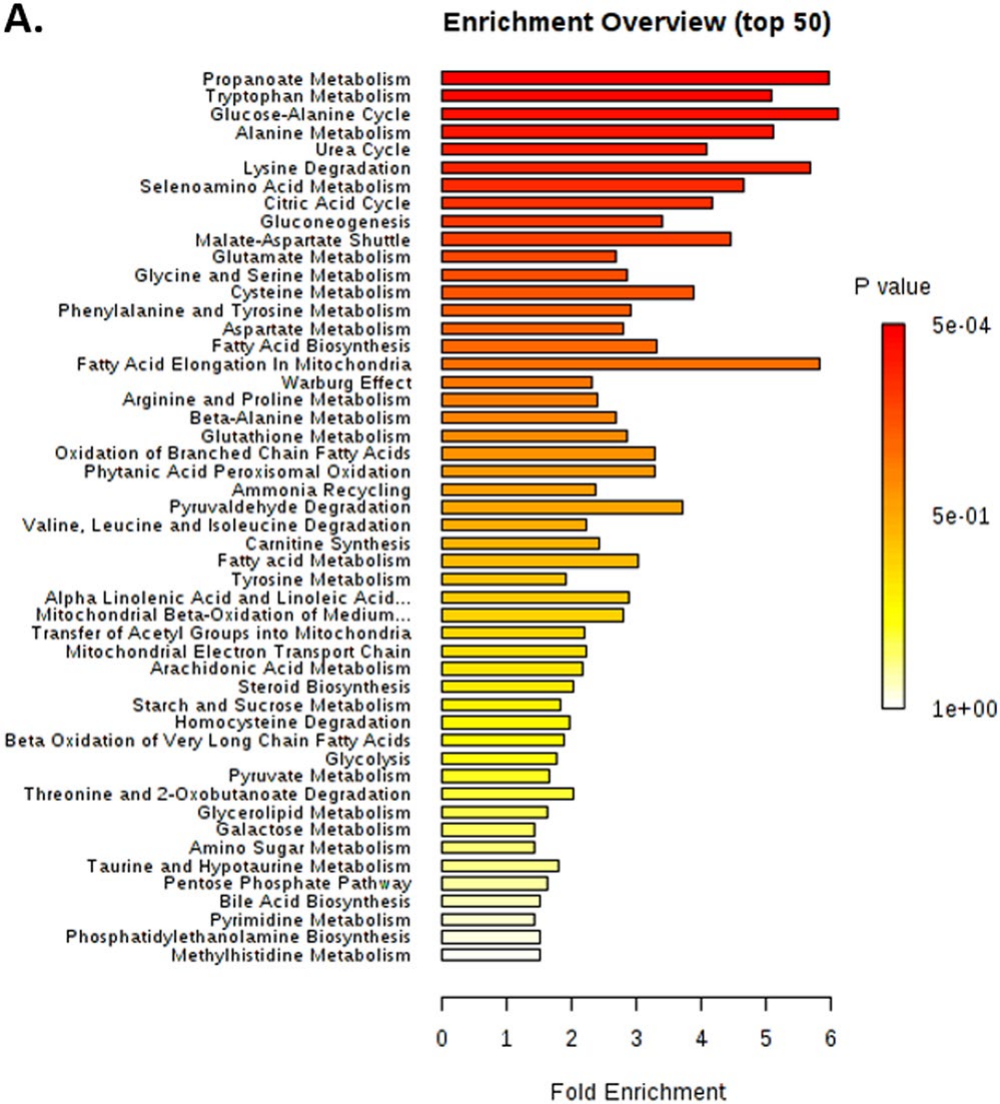
Enrichment analysis showed significantly altered metabolic pathways in young CAP. (**A**) Enrichment analysis of metabolites based on pathway-associated metabolite sets. The strongest association was found in propanoate and tryptophan metabolism.

Since differentially abundant metabolites and altered ratios were detected, we then asked whether the altered metabolites played a crucial role in their respective metabolic pathways. To this end, enrichment analysis was performed to identify metabolite sets grouped together by their involvement in the same biological phenotypes (Fig. 3A). The enrichment analysis results as well as P value and FDR for significantly regulated pathways based on pathway-associated metabolite sets are summarized in Table 3. In biological processes, the strongest association was found in propanoate and tryptophan metabolism (Table 3), which correlated with the respective metabolite ratio alteration (Table 2). However, additional clinical trials based on larger cohorts are still warranted to test the reliability of this pathway analysis.

**Table 3:** Enrichment analysis of the differentially expressed metabolites based on pathway-associated metabolite sets.

Metabolite Set	Total	Hits	Statistic	P value	FDR
Propanoate Metabolism	42.00	8.00	12.42	0.00	0.00
Tryptophan Metabolism	60.00	8.00	10.58	0.00	0.00
Glucose-Alanine Cycle	13.00	5.00	12.71	0.00	0.00
Alanine Metabolism	17.00	6.00	10.67	0.00	0.00
Urea Cycle	29.00	12.00	8.49	0.00	0.00
Lysine Degradation	30.00	5.00	11.87	0.00	0.00
Selenoamino Acid Metabolism	28.00	4.00	9.71	0.00	0.01
Citric Acid Cycle	32.00	6.00	8.70	0.00	0.02
Gluconeogenesis	35.00	7.00	7.12	0.00	0.02
Malate-Aspartate Shuttle	10.00	4.00	9.27	0.00	0.03
Glutamate Metabolism	49.00	13.00	5.61	0.00	0.03
Glycine and Serine Metabolism	59.00	17.00	5.96	0.00	0.03
Cysteine Metabolism	26.00	5.00	8.09	0.00	0.03
Phenylalanine and Tyrosine Metabolism	28.00	9.00	6.08	0.01	0.03
Aspartate Metabolism	35.00	12.00	5.86	0.01	0.04
Fatty Acid Biosynthesis	35.00	6.00	6.92	0.01	0.04
Fatty Acid Elongation In Mitochondria	35.00	1.00	12.17	0.01	0.06
Warburg Effect	58.00	12.00	4.82	0.01	0.06
Arginine and Proline Metabolism	53.00	14.00	5.02	0.02	0.06
Beta-Alanine Metabolism	34.00	8.00	5.60	0.02	0.06
Glutathione Metabolism	21.00	6.00	5.99	0.02	0.06
Oxidation of Branched Chain Fatty Acids	26.00	3.00	6.88	0.02	0.06
Phytanic Acid Peroxisomal Oxidation	26.00	3.00	6.88	0.02	0.06
Ammonia Recycling	32.00	10.00	4.95	0.02	0.06
Pyruvaldehyde Degradation	10.00	2.00	7.76	0.02	0.08
Valine, Leucine and Isoleucine Degradation	60.00	10.00	4.66	0.04	0.13
Carnitine Synthesis	22.00	4.00	5.10	0.04	0.14
Fatty acid Metabolism	43.00	2.00	6.35	0.04	0.14
Tyrosine Metabolism	72.00	10.00	4.03	0.04	0.14
Alpha Linolenic Acid and Linoleic Acid Metabolism	19.00	3.00	6.01	0.05	0.14

### Dysregulated metabolites were correlated with clinical inflammatory markers in young CAP patients

**Figure 4:**
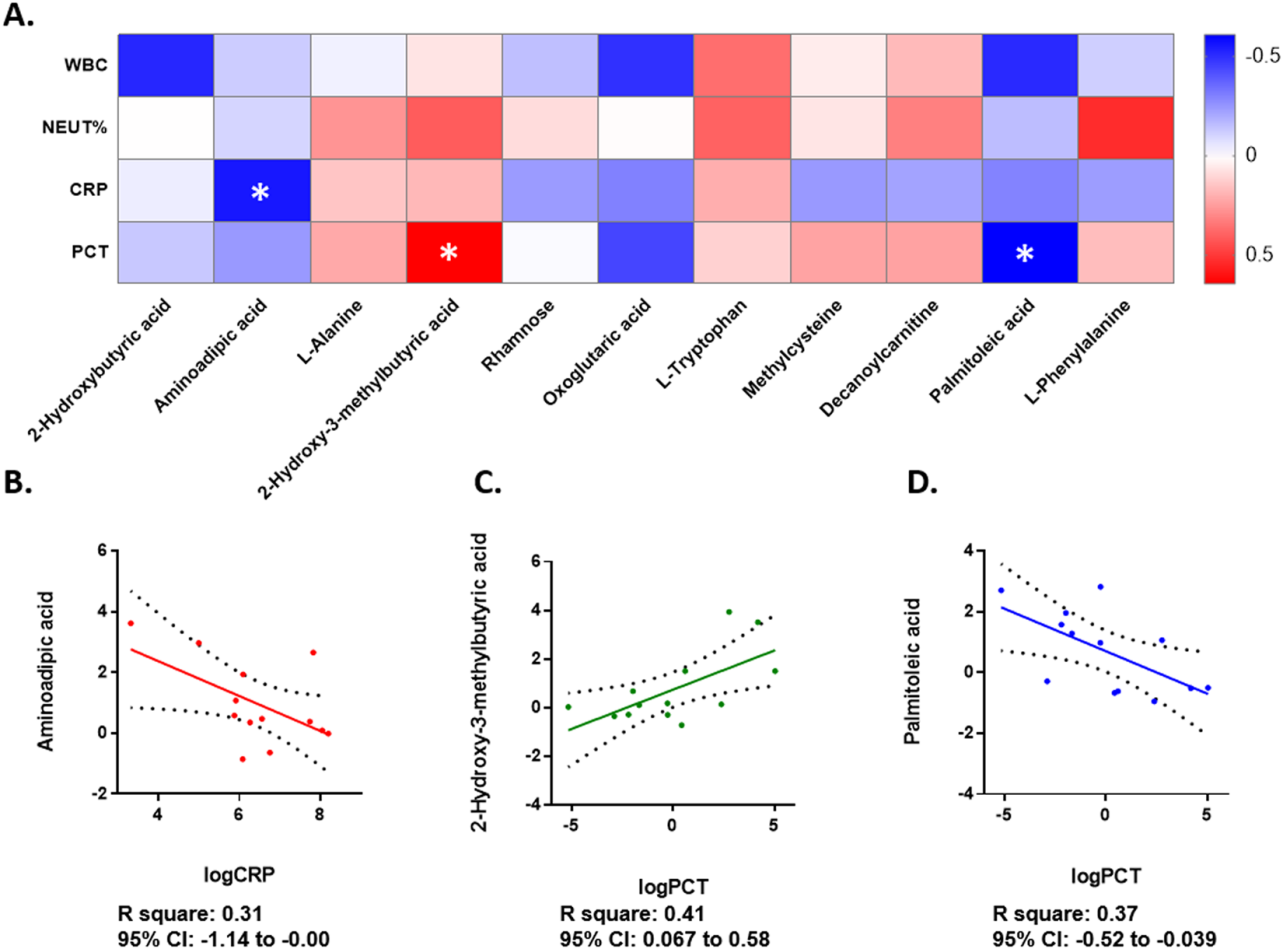
Dysregulated metabolites were correlated to clinical inflammatory markers in young CAP. (**A**) Correlations between CRP, PCT, neutrophil percentage and WBC count and altered metabolites and ratios in young CAP adults. The colors in the heatmap stood for efficiency of the Pearson’s correlation. *p < 0.05, **p < 0.01. (**B**) Simple linear regression model with log 2 transformed CRP and relative serum Aminoadipic acid levels. The y axis represented serum Aminoadipic acid level, and the x axis represented log 2 transformed CRP. R square was 0.31, and 95% CI was −1.14 to −0.00. (**C**) Simple linear regression model with log 2 transformed PCT and relative serum 2-Hydroxy-3-methylbutyric acid level. The y axis represented serum 2-Hydroxy-3-methylbutyric acid level, and the x axis represented log 2 transformed PCT. R square was 0.41, and 95% CI was 0.067 to 0.58. (**D**) Simple linear regression model with log 2 transformed PCT and relative serum Palmitoleic acid level. The y axis represented serum Palmitoleic acid level, and the x axis represented log 2 transformed PCT. R square was 0.37, and 95% CI was −0.52 to −0.039.

To further employ the relationship between the alterations in metabolites and clinical features upon patient admission to the hospital, differential metabolite correlations with clinical inflammatory markers, including CRP, PCT, neutrophil percentage and WBC count, in young CAP patients were conducted afterwards (Fig. 4A). CRP and PCR were log 2 transformed for normal distribution. Aminoadipic acid was found to be significantly negatively correlated with CRP levels (Fig. 4B); 2-hydroxy-3-methylbutyric acid positively (Fig. 4C); and palmitoleic acid negatively (Fig. 4D) correlated with PCT levels. The correlation between metabolites and clinical inflammatory markers indicated potential diagnostic value for inflammation.

### Diagnostic value of the metabolites by ROC analysis for young CAP patients

**Figure 5:**
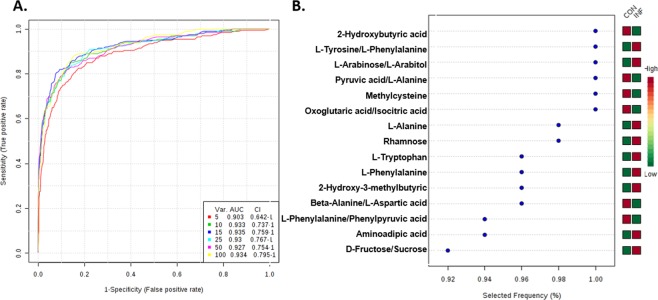
Diagnostic value for young CAP of the metabolites by multivariate ROC analysis. (**A**) Multivariate Exploratory ROC Analysis generated by Monte-Carlo cross validation (MCCV) using balanced sub-sampling. Linear SVM were utilized for classification model and features were ranked by SVM built-in. (**B**) Significant features of metabolites and ratios in multivariate Exploratory ROC Analysis.

Finally, to further identify the diagnostic value of the altered metabolites and ratios, receiver operating characteristic (ROC) analysis was performed. The differentially abundant metabolites and altered ratios with significant diagnostic values are shown in Table 4. The top 3 metabolites of diagnostic values are 2-Hydroxybutyric acid(AUC = 0.90), Methylcysteine(AUC = 0.85), and L-Alanine(AUC = 0.84). The AUC for CRP and PCT are 0.93 and 0.91 respectively. The alteration and ROC analysis in circulatory amino acids indicated that amino acid levels might be a potential diagnostic marker for CAP; however, further validation analyses based on larger cohorts are warranted. Multivariate ROC curve-based exploratory analyses were together performed for automated important feature identification and performance evaluation. Linear SVMs were utilized for the classification model, and features were ranked by built-in SVMs. ROC curves based on the cross validation (CV) performance and the respective P value, AUC and confidence interval are shown in Fig. 5A, and the top 15 significant features ranked by frequencies of being selected are shown in Fig. 5B.

**Table 4:** Diagnostic value of the metabolites by ROC analysis.

Name	AUC	T-tests	Log2 FC
**Diagnostic value of the metabolites**			
2-Hydroxybutyric acid	0.90	0.00	−1.46
Methylcysteine	0.85	0.00	1.31
L-Alanine	0.84	0.00	1.22
Aminoadipic acid	0.83	0.00	−1.57
L-Tryptophan	0.82	0.00	0.72
2-Hydroxy-3-methylbutyric acid	0.79	0.00	−1.07
L-Phenylalanine	0.76	0.00	−0.46
Oxoglutaric acid	0.73	0.00	−0.82
Decanoylcarnitine	0.71	0.00	−0.97
**Diagnostic value of the metabolites ratio**			
Ratio of Pyruvic acid/L-Alanine	0.94	0.00	−1.99
Ratio of L-Tyrosine/L-Phenylalanine	0.87	0.00	0.66
Ratio of L-Arabinose/L-Arabitol	0.86	0.00	−1.01
Ratio of Oxoglutaric acid/Isocitric acid	0.82	0.00	−1.27
Ratio of L-Phenylalanine/Phenylpyruvic acid	0.79	0.00	−1.23
Ratio of Ornithine/L-Arginine	0.73	0.00	−0.78
**Diagnostic value of CRP and PCT**			
CRP	0.93	0.00	−6.88
PCT	0.91	0.00	−4.96

## Discussion

In this study, we investigated the global metabolomic profile in young CAP patients. Of note, our results showed that serum metabolites and their ratio with biological significance were altered in young adults with CAP, which were enriched mainly in propanoate and tryptophan metabolism, as well as antibiotic-associated pathways. Altered metabolites were correlated with clinical inflammatory markers and were of great diagnostic value for CAP.

In agreement with our results, a recent study proved that multiple nucleic acid metabolites were increased in the early sepsis of non-survivors compared with those in survivors, indicating potentially differential metabolic processes in early sepsis (bile acid metabolism, protein catabolism, inflammation, and oxidative stress)^6,7^. The present analysis provides evidence that even in young adults with mild CAP who are cured by moxifloxacin therapy, inflammation caused by lung infection is strongly associated with metabolic changes in the disease setting, demonstrating the potential diagnostic value of metabolomics signatures to diagnose, evaluate inflammation severity and direct therapeutic strategies for CAP. The differentially abundant metabolites and their ratios could be novel biochemical markers for young adults with CAP, although quantification and validation are necessary in studies with larger cohorts.

The differentially abundant metabolites in young adults with CAP are associated with mainly amino acid metabolism, which is correlated to the pathophysiological mechanism. CAP in young adults is caused mainly by *Streptococcus pneumoniae*, a gram-positive diplococcus with a well-formed capsule. The growth of pneumococci in the host depends on the catabolism of utilized carbon sources. It has been proven that glucose dissimilation could result in pyruvate via the Embden-Meyerhof-Parnas (EMP) pathway, which is the key for metabolism in *Streptococcus pneumoniae*^6,7^. In our study, amino acids, including L-alanine, methylcysteine, L-phenylalanine, aminoadipic acid and L-tryptophan, were markedly dysregulated in the serum of young CAP patients and are also involved in the pathways of amino acid biosynthesis in *Streptococcus pneumoniae*. In accordance with our results, a recent study also provides an overview of metabolic changes occurring in the infectious parapneumonic effusions caused by pneumococci^6,7^, indicating that pneumococci may lead to the aggressive metabolic processes of glucose consumption and subsequent biosynthesis of amino acids.

The major novelty of the present study is that we first studied the global metabolic profile in young adults with CAP who could be cured by moxifloxacin therapy afterwards. Although it is well established that metabolomic profile alterations are common in severe lung infection, especially in sepsis, the metabolism change in young adults with CAP has not been evaluated before^6,7^, limiting the predictive and therapeutic values of metabolites for those patients. The present findings point to the utility of non-targeted metabolic profile surveys for identifying biochemical markers for young adults with CAP. In addition, the correlation between metabolites and clinical inflammatory markers, including CRP and PCT, also provides new perspectives on infection severity evaluation. It is noteworthy that for young adults with CAP, although the symptoms are normally mild and patients recover with routine antibiotic treatments, untimely diagnosis and treatments might give rise to devastating complications and outcomes. Therefore, the identification of an evaluation method using metabolomics could be helpful for improving the diagnosis and prognosis of young adults with mild CAP.

The limitation of this study is the small sample size and relatively low sensitivity of metabolomics analysis, but this study provides the advantage of accurately studying a wide range of metabolites associated with bacterial and cellular pathways. Moreover, serum metabolite measurements were based on non-targeted metabolic methods, which restricted precise evaluation. Thus, we suggest that our findings warrant further study for targeted metabolomics confirmation. The present study can be considered only an exploratory study. Therefore, further large population-based studies are necessary to further verify the accuracy of serum metabolites as novel markers to diagnose CAP.

## Conclusion

The current work established the biomarker function of serum metabolites for the diagnosis and evaluation of young patients with CAP. Altered serum metabolites and their ratio with biological significance were enriched mainly in propanoate and tryptophan metabolism, as well as antibiotic-associated pathways. From this perspective, our data suggest that global metabolomics provides potential circulatory markers for diagnosing, evaluating and treating young adults with CAP.

## Supplementary information

Supplementary information.

## Data Availability

Data and material are available upon request.

## References

[1] Prina E, Ranzani OT, Torres A (2015). Community-acquired pneumonia. Lancet.

[2] Cilloniz C (2016). Community-acquired pneumonia related to intracellular pathogens. Intensive care medicine.

[3] Asai, N. *et al*. Efficacy and accuracy of qSOFA and SOFA scores as prognostic tools for community-acquired and healthcare-associated pneumonia. *International journal of infectious diseases: IJID: official publication of the International Society for Infectious Diseases*, 10.1016/j.ijid.2019.04.020 (2019). 10.1016/j.ijid.2019.04.020 31028877

[4] Leoni D, Rello J (2017). Severe community-acquired pneumonia: optimal management. Current opinion in infectious diseases.

[5] Lippi G, Meschi T, Cervellin G (2011). Inflammatory biomarkers for the diagnosis, monitoring and follow-up of community-acquired pneumonia: clinical evidence and perspectives. European journal of internal medicine.

[6] Chiu CY (2016). Metabolomic Profiling of Infectious Parapneumonic Effusions Reveals Biomarkers for Guiding Management of Children with Streptococcus pneumoniae Pneumonia. Scientific reports.

[7] Seymour CW (2013). Metabolomics in pneumonia and sepsis: an analysis of the GenIMS cohort study. Intensive care medicine.

[8] Arshad H (2019). Decreased plasma phospholipid concentrations and increased acid sphingomyelinase activity are accurate biomarkers for community-acquired pneumonia. J Transl Med.

[9] Muller DC (2019). Phospholipid levels in blood during community-acquired pneumonia. PLoS One.

[10] Torres A (2019). Challenges in severe community-acquired pneumonia: a point-of-view review. Intensive care medicine.

[11] She J (2018). Correlation of Triiodothyronine Level with In-Hospital Cardiac Function and Long-Term Prognosis in Patients with Acute Myocardial Infarction. Disease Markers.

[12] She J (2017). Hemoglobin A1c is associated with severity of coronary artery stenosis but not with long term clinical outcomes in diabetic and nondiabetic patients with acute myocardial infarction undergoing primary angioplasty. Cardiovascular diabetology.

[13] She J (2018). Targeting amino acids metabolic profile to identify novel metabolic characteristics in atrial fibrillation. Clinical science.

[14] She J (2018). Correlation of Triiodothyronine Level with In-Hospital Cardiac Function and Long-Term Prognosis in Patients with Acute Myocardial Infarction. Dis Markers.

[15] Qiu Y (2009). Serum metabolite profiling of human colorectal cancer using GC-TOFMS and UPLC-QTOFMS. Journal of proteome research.

[16] Wang JH (2013). Prognostic significance of 2-hydroxyglutarate levels in acute myeloid leukemia in China. Proceedings of the National Academy of Sciences of the United States of America.

[17] Ni Y (2012). ADAP-GC 2.0: deconvolution of coeluting metabolites from GC/TOF-MS data for metabolomics studies. Analytical chemistry.

[18] Xia J, Wishart DS (2016). Using MetaboAnalyst 3.0 for Comprehensive Metabolomics Data Analysis. Current protocols in bioinformatics.

[19] Xia J, Sinelnikov IV, Han B, Wishart DS (2015). MetaboAnalyst 3.0–making metabolomics more meaningful. Nucleic acids research.

[20] Xia J, Mandal R, Sinelnikov IV, Broadhurst D, Wishart DS (2012). MetaboAnalyst 2.0–a comprehensive server for metabolomic data analysis. Nucleic acids research.

[21] Xia J, Wishart DS (2011). Web-based inference of biological patterns, functions and pathways from metabolomic data using MetaboAnalyst. Nature protocols.

[22] Xia J, Wishart DS (2011). Metabolomic data processing, analysis, and interpretation using MetaboAnalyst. Current protocols in bioinformatics.

[23] Xia J, Psychogios N, Young N, Wishart DS (2009). MetaboAnalyst: a web server for metabolomic data analysis and interpretation. Nucleic acids research.

[24] Xia J, Broadhurst DI, Wilson M, Wishart DS (2013). Translational biomarker discovery in clinical metabolomics: an introductory tutorial. Metabolomics: Official journal of the Metabolomic Society.

[25] Xia J, Sinelnikov IV, Wishart DS (2011). MetATT: a web-based metabolomics tool for analyzing time-series and two-factor datasets. Bioinformatics.

[26] Xia J, Wishart DS (2010). MetPA: a web-based metabolomics tool for pathway analysis and visualization. Bioinformatics.

[27] Xia J, Wishart DS (2010). MSEA: a web-based tool to identify biologically meaningful patterns in quantitative metabolomic data. Nucleic acids research.

[28] Frolkis A (2010). SMPDB: The Small Molecule Pathway Database. Nucleic acids research.

[29] Jewison T (2014). SMPDB 2.0: big improvements to the Small Molecule Pathway Database. Nucleic acids research.

