# Supplementary material for: Serum metabolite profiles as potential biochemical markers in young adults with community-acquired pneumonia cured by moxifloxacin therapy
Source: Sci Rep. 2020 Mar 10;10:4436. doi: 10.1038/s41598-020-61290-x (PMC7064523; doi:10.1038/s41598-020-61290-x)

Supplementary Information

Serum metabolite profiles as potential biochemical markers in young adults with community acquired pneumonia cured by moxifloxacin therapy.

Bo Zhou [1], Bowen Lou[2,4], Junhui Liu[3#], Jianqing She[2,4#]

[1]Respiratory and critical care medicine, First Affiliated Hospital of Medical College, Xi'an Jiaotong University, Xi'an, 710048

[2] Cardiology Department, First Affiliated Hospital of Medical College, Xi'an Jiaotong University, Xi'an, 710048

[3] Diagnostic Department, First Affiliated Hospital of Medical College, Xi'an Jiaotong University, Xi'an, 710048

[4] Key Laboratory of Environment and Genes Related to Diseases, Ministry of Education, Xi'an 710048

*To whom correspondence should be addressed:

Jianqing She, E-mail: jianqingshe@xjtu.edu.cn,

Junhui Liu, E-mail: liu1109@xjtu.edu.cn;

Supplementary Figure1: Data normalization.

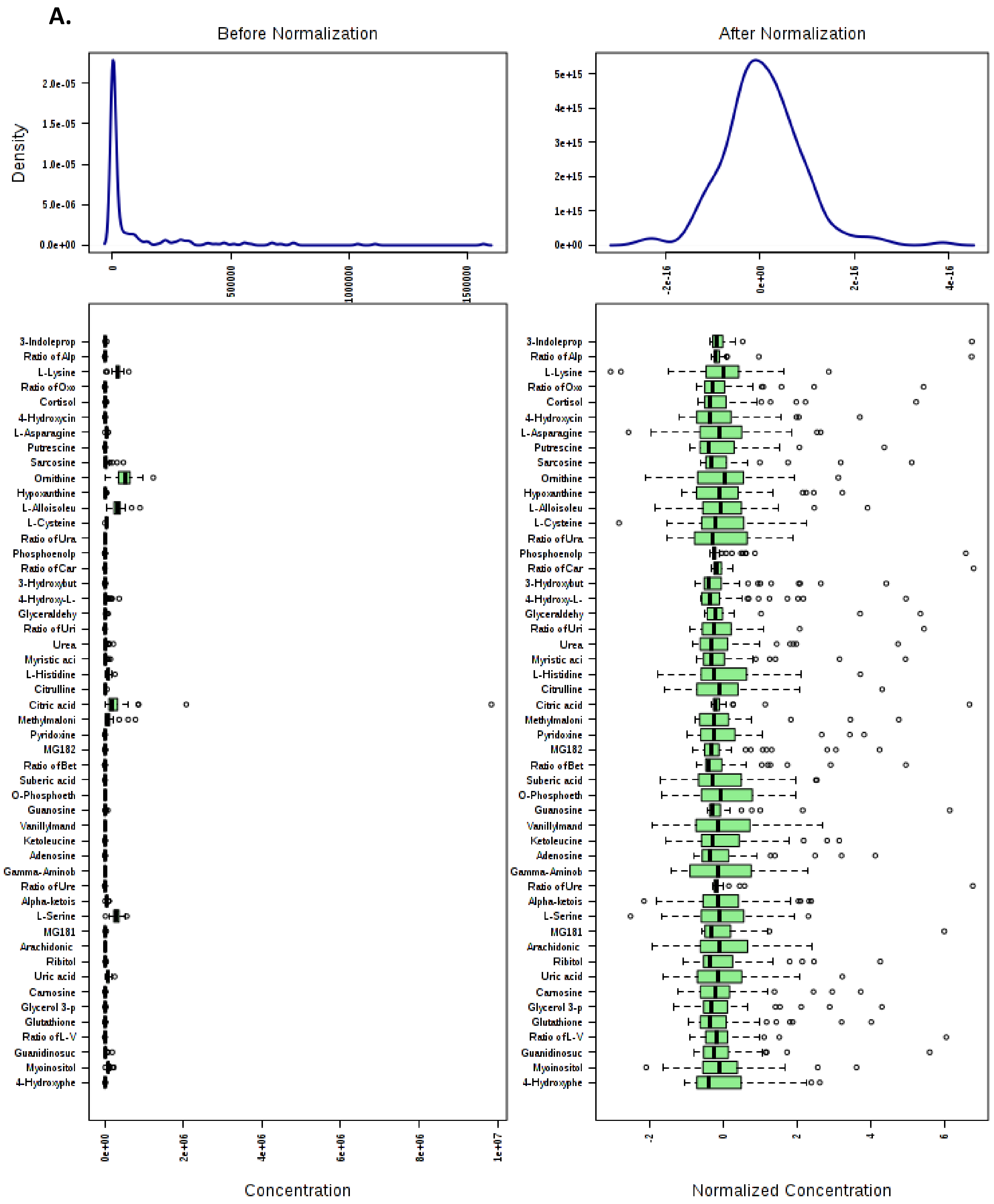

Supplementary Figure2: Heatmap showing metabolites classes in young CAP as compare to control.

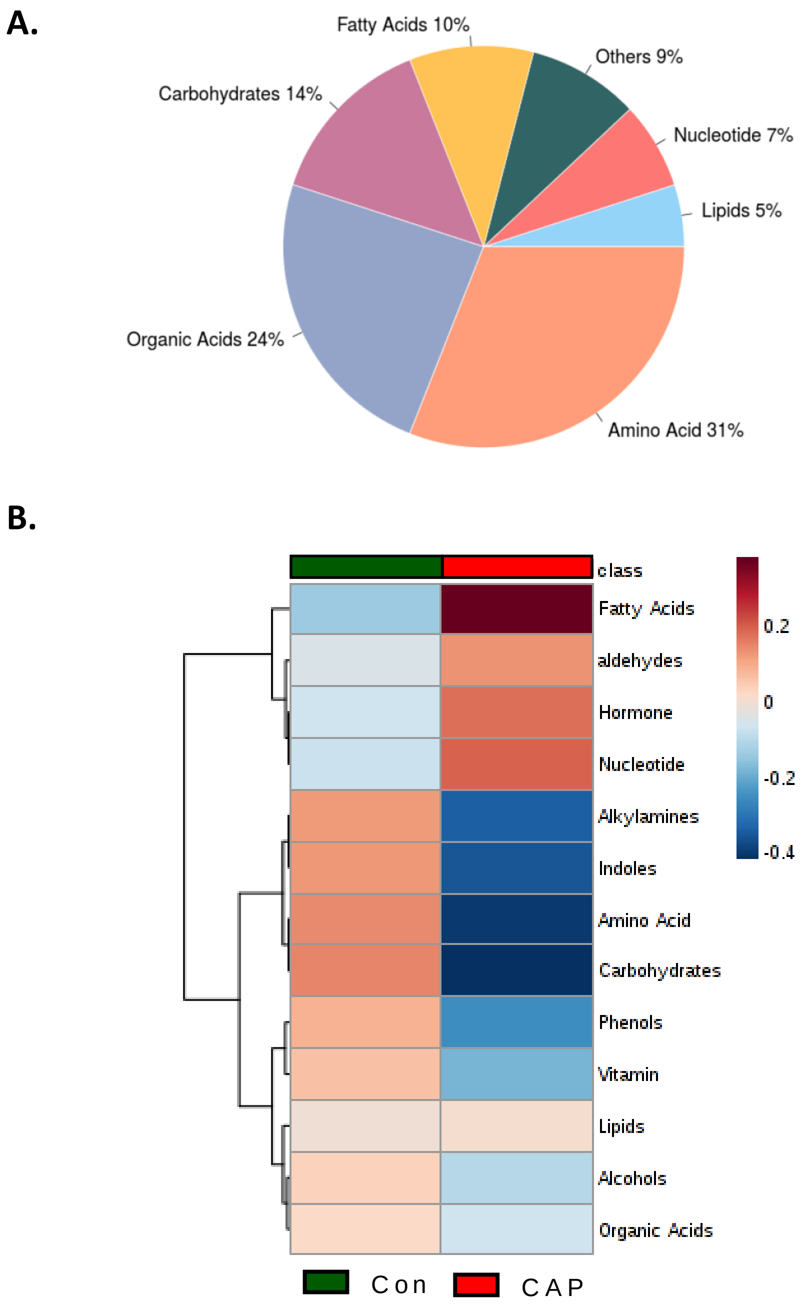

Supplement: Supplementary file 1 — Supplementary information. [file 41598_2020_61290_MOESM1_ESM.pdf]
